# *Bacillus coagulans* Alleviates Intestinal Damage Induced by TiO_2_ Nanoparticles in Mice on a High-Fat Diet

**DOI:** 10.3390/foods11213368

**Published:** 2022-10-26

**Authors:** Qingying Shi, Chen Yang, Bingjie Zhang, Dongxiao Chen, Fuping Lu, Huabing Zhao

**Affiliations:** 1 College of Biotechnology, Tianjin University of Science and Technology, 9 TEDA 13th Street, Tianjin 300457, China; 2 Key Laboratory of Industrial Fermentation Microbiology, Ministry of Education, Tianjin University of Science and Technology, Tianjin 300450, China

**Keywords:** TiO_2_ NPs, *Bacillus coagulans*, intestinal microorganisms, metabolism, high-fat diet

## Abstract

Titanium dioxide nanoparticles (TiO_2_ NPs) are generally added in considerable amounts to food as a food additive. Oral exposure to TiO_2_ NPs could induce intestinal damage, especially in obese individuals with a high-fat diet. The probiotic *Bacillus coagulans* (*B. coagulans*) exhibits good resistance in the gastrointestinal system and is beneficial to intestinal health. In this study, *B. coagulans* was used to treat intestinal damage caused by TiO_2_ NPs in high-fat-diet mice via two intervention methods: administration of TiO_2_ NPs and *B. coagulans* simultaneously and administration of TiO_2_ NPs followed by that of *B. coagulans*. The intervention with *B. coagulans* was found to reduce the inflammatory response and oxidative stress. A 16S rDNA sequencing analysis revealed that *B. coagulans* had increased the diversity of gut microbiota and optimized the composition of gut microbiota. Fecal metabolomics analysis indicated that *B. coagulans* had restored the homeostasis of sphingolipids and amino acid metabolism. The intervention strategy of administering TiO_2_ NPs followed by *B. coagulans* was found to be more effective. In conclusion, *B. coagulans* could alleviate intestinal damage induced by TiO_2_ NPs in high-fat-diet mice TiO_2_ *B. coagulans*. Our results suggest a new avenue for interventions against intestinal damage induced by TiO_2_ NPs.

## 1. Introduction

Titanium dioxide nanoparticles (TiO_2_ NPs) are promising nanomaterials that have been widely used in a variety of fields, such as the cosmetic industry, the field of medicine, and the food industry [1]. TiO_2_ NPs are generally added in considerable amounts to food, as colorants to improve the sensory properties of food and as food preservatives due to their antibacterial properties [2]. For example, sweets and candies contain high levels of TiO_2_ NPs, even as much as 2.5 mg of titanium per g of food [1,3]. A vital way for people to consume TiO_2_ NPs is orally. It is estimated that the dietary consumption of TiO_2_ NPs was about 2–3 mg/kg/day for children and 1 mg/kg/day for adults in the United Kingdom (UK) [4]. It is worth noting that the Food and Drug Administration (FDA) allows TiO_2_ NPs to be used as a food additive in the United States, but its content cannot exceed 1% of the total quantity of the product [5]. In addition, in 2021, the European Food Safety Agency (EFSA) issued a safety assessment of TiO_2_ NPs, indicating that TiO_2_ NPs are no longer considered safe as a food additive [6].

Numerous studies have documented intestinal damage, as, after ingestion, TiO_2_ NPs stay for extended periods in the intestinal tract and interact with intestinal microorganisms and intestinal epithelial cells [7,8,9,10]. Gut microbiota is an ecosystem with complex interactions, which plays a vital role in human health. The intestinal epithelial barrier can protect the body from commensal bacteria, pathogens, and foreign particles. It is believed that oxidative stress plays a vital role in TiO_2_ NP toxicityTiO_2_, and the increase in the oxidative stress level has been shown to lead to disturbed serum biochemical parameters and elevated inflammatory factor levels [11,12]. Ruiz et al. indicated that TiO_2_ NPs aggravate colitis by increasing the production of reactive oxygen species (ROS) and inflammasomes [13]. Moreover, our previous study presented that exposure to TiO_2_ NPs affects the intestinal microecology in mice: for example, gut microbiota diversity and composition, short-chain fatty acid (SCFA) production, inflammatory response levels, and gut-associated metabolism [10].

Some high-fat foodsTiO_2_, such as chocolates, puffed foods, jams, and candies, contain large amounts of TiO_2_ NPs. Obese people who consume large amounts of high-fat foods can be exposed to more TiO_2_ NPs, which may cause more pronounced health effects. Moreover, obese populations have been shown to be more vulnerable to the potentially harmful effects of TiO_2_ NPs. Cao et al. found that the effects of TiO_2_ NPs, such as intestinal microbial disorders, decreased levels of SCFAs, abnormal changes in cytokines, and negative histopathology, were greater on obese mice on a high-fat diet compared to non-obese mice [14]. As per one study, about 1.9 billion adults worldwide are overweight, and more than 600 million people are obese [15]. Considering that obesity is still increasing globally and has become a major socio-economic burden [16], the impact of TiO_2_ NPs on obese individuals deserves attention, and it is important to actively seek corresponding preventive or therapeutic intervention programs.

*Bacillus coagulans* (*B. coagulans*), with good heat resistance, strong resistance to acids, and a high survival rate after reaching the gastrointestinal system, has been reported as safe by the FDA and the EFSA and included in the Generally Recognized As Safe (GRAS) and Qualified Presumption of Safety (QPS) lists [17]. Microbial preparations made from *B. coagulans* have been widely used in food, medicine, animal husbandry, etc., enjoying broad application prospects. B.Coagulans MTCC 5856, a kind of *Bacillus coagulans*, has been found to exhibit excellent resistance to gastric acid and can withstand high temperatures of up to 90 °C [18]. Tanvi et al. [19] used *B. coagulans* MTCC 5856 to intervene in mice with inflammatory bowel disease (IBD) and found that *B. coagulans* MTCC 5856 has the ability to maintain intestinal epithelial integrity, promote SCFA production, and reduce colonic inflammation.

Here, taking into consideration its good properties, *Bacillus coagulans* was employed as a probiotic to treat intestinal damage caused by TiO_2_ NPs. A mouse model of obesity induced by a high-fat diet was established in this study. These obese mice were fed with TiO_2_ NPs daily for eight weeks. Two kinds of intervention strategies with *Bacillus coagulans* were conducted to explore both the regulatory effect of *Bacillus coagulans* and the influence of intervention methods on its effectiveness. This study will provide new insights into the prevention of the negative effects of TiO_2_ NPs on people with chronic diseases.

## 2. Materials and Methods

### 2.1. Animals and Treatments

Six-week-old C57BL/6J mice (male, weight 20–24 g) were purchased from SPF (Beijing, China) Biotechnology Co., Ltd. All animal experiments were approved by the Animal Ethics Committee of JAK BIO company (JKX-2106-01). The animals were kept in a temperature-controlled room (25 °C, 45% humidity) exposed to a 12 h/12 h dark/light cycle and had free access to water and food. After a 1-week adaptation period, the mice were randomly divided into a normal-diet-fed group (5 kcal%) and a high-fat-diet-fed group (60 kcal%). The high-fat-diet mice were further divided into four subgroups (*n* = 5). One subgroup was given only a high-fat diet for eight weeks, one subgroup was subjected to a high-fat diet containing TiO_2_ NPs for eight weeks, one subgroup was fed a high-fat diet containing TiO_2_ NPs and *B. coagulans* MTCC 5856 simultaneously for eight weeks, and one subgroup was given a high-fat diet containing TiO_2_ NPs for four weeks followed by *B. coagulans* MTCC 5856 for four weeks. Table 1 presents the specific groupings. TiO_2_ NPs equivalent to 0.2% of the body weight of the mice were fed incorporated in a high-fat diet, a dose corresponding to one-fifth of the allowable maximum daily intake prescribed by the FDA and 50 times the estimated average daily human intake [14]. The *B. coagulans* MTCC 5856 was administered as a gavage daily with 10^9^ CFUs suspended in 1 mL of the medium to the mice of the treatment groups, and an equal volume of normal saline was gavaged daily to the mice of the control group, and the HFD group. All the mice were weighed weekly, and the colorectal lengths of the mice were also measured. The mice were sacrificed by cervical dislocation after eight weeks, and the appropriate tissues were harvested.

The probiotic bacterium *B. coagulans* MTCC 5856 used in this study is a patented strain of Sabinsa Corporation/Sami Labs Limited and deposited to Microbial Type Culture Collection and Gene Bank (MTCC), Chandigarh, India. *B. coagulans* MTCC 5856 was manufactured by Sami Labs Limited as per a proprietary method in a good manufacturing practices (GMP) facility in Bangalore, India. *B. coagulans* MTCC 5856 spores were administered as a gavage daily with 10^9^ CFU suspended in 1 mL of medium to the mice in this study, a dose corresponding to the safe and tolerable level for human and effective in diarrhea-predominant irritable bowel syndrome patients according to previous research [20,21,22]. A viable spore count of *B. coagulans* MTCC 5856 was determined as per the method described previously [23]. Briefly, 1.0 g of *B. coagulans* MTCC 5856 was mixed in sterile saline (0.9% NaCl, *w*/*v*) and then incubated in a water bath for 30 min at 75 °C, followed by immediate cooling to below 45 °C. The suspension was further serially diluted in sterile saline, and the viable count was enumerated by plating on glucose yeast extract agar by pour plate method. The plates were incubated at 37 °C for 48–72 h. Analysis was performed twice in triplicate. The average means of viable spore counts were expressed in cfu/g.

### 2.2. Measurement of the Blood Glucose

The blood glucose in the tail vein blood of the mice was measured by a blood glucose meter (Sinocare Inc., Changsha, China).

### 2.3. Analysis of the Lipid Profiles in Serum

The blood samples were collected by eyeball extraction and then transferred into 1.5 mL microcentrifuge tubes. After the blood samples were centrifuged at 800× *g* for 15 min, the levels of total cholesterol (TC) and triglyceride (TG) in the upper layer serum were assessed using the TC assay kit and the TG assay kit (Nanjing Jiancheng Bioengineering Institute, Nanjing, China) according to the manufacturer’s instructions.

### 2.4. Assessment of Pro-Inflammatory Cytokines

To determine the inflammatory responses, the expression levels of interleukin 1β (IL-1β), interleukin 6 (IL-6), and tumor necrosis factor (TNF-α) in serums and colon tissues of the mice were measured by using enzyme-linked immunosorbent assay (ELISA) kits (Shanghai Enzyme-linked Biotechnology Co., Ltd., Shanghai, China).

### 2.5. Evaluation of Antioxidant Enzyme

The oxidative stress response was investigated. The total antioxidant capacity assay kit (T-Aoc), and the superoxide dismutase (SOD) assay kit (Nanjing Jiancheng Bioengineering Institute, Nanjing, China) were used to detect the levels of T-Aoc and SOD in the sera and colon tissues of the mice according to the manufacturer’s instructions.

### 2.6. Analysis of Gut Microbiota

Before the mice were sacrificed, their individual fecal samples were collected and placed in 1.5 mL sterilized tubes. Then, all the samples were snap-frozen on dry ice and stored at −80 °C. The fecal genomic DNA was extracted using a fecal genomic DNA extraction kit (Tiangen Biotech Co., Ltd., Beijing, China). The Illumina HiSeq platform (Nuohe Zhiyuan Bio-Information Technology Co., Ltd., Tianjin, China) was used to analyze the 16S ribosomal RNA genes (16S rRNA) in the fecal samples. Reactions were conducted in triplicate, and the V3-V4 region of genomic DNA was amplified using the specific primers 341F (5′-ACTCCTACGGGAGGCAGCAG-3′) and 806R (5′-GGACTACHVGGGTWTCTAAT-3′). The obtained data were analyzed with the help of QIME (Version 1.8.0) and R (Version 4.0.5) software packages.

### 2.7. Quantification of Short-Chain Fatty Acids

In the cecal contents of mice, the SCFAs, including acetic acid (AA), propionic acid (PA), butyric acid (BA), valeric acid (VA), and isovaleric acid (IVA), were detected by gas chromatography-mass spectrometry (GC–MS, Agilent, Santa Clara, CA, USA). Briefly, cecal contents (0.1 g) were suspended in 0.1 mL of 20% phosphoric acid solution, homogenized adequately for 2 min by vortex, and centrifuged for 10 min at 14,500× *g*. After centrifugation, the supernatants were extracted with 500 μL of ethyl acetate and centrifuged for 10 min at 14,500× *g*. 4-Methylvaleric acid at a final concentration of 500 µM was added as the internal standard. The coefficient of determination for a standard curve, which included five concentrations, 0.05, 0.10, 0.15, 0.20, and 0.25 μL/mL, was greater than 0.99. The key parameters for GC–MS analysis are shown in Table S1 in Supplementary Information. The Agilent Mass Hunter software was used to process the data.

### 2.8. Metabolomic Analysis

Approximately 20 mg of each fecal sample was weighed and added to 400 µL of pre-cooled MeOH/H_2_O (8/2, *v*/*v*) buffer. The samples were subjected to ultrasonic for 10 min and allowed to stand for 30 min at −20 °C. Then, after centrifugation at 12,400× *g* for 10 min at 4 °C, 300 µL of the supernatants were transferred into new tubes. After further centrifugation at 12,400× *g* for 3 min at 4 °C, 200 µL of the supernatants were placed into liner pipes as the test solutions.

The specific detection information is presented in Table S2 and Table S3 in Supplementary Information. The obtained raw data were converted into mzML format by ProteoWizard software; and the XCMS program was used for peak extraction, alignment, and retention time correction. The peak areas were corrected using the SVR method, and peaks with deletion rates  >  50% were filtered from each group of samples. The metabolites were identified by matching the information with the metabolic database. Further statistical analysis was performed by MetaboAnalyst.

### 2.9. Statistical Analysis

Statistical analysis was performed using the SPSS statistical software (version 26.0). Multiple comparisons were made using a one-way analysis of variance (ANOVA). A nonparametric Kruskal–Wallis test was applied to analyze the statistical differences for the data that had failed the normality test. The web-based platform MetaboAnalyst 5.0 (https://www.metaboanalyst.ca/ (accessed on 21 March 2022)) was used to perform metabolic pathway analysis of the differentially expressed metabolites. All the samples were analyzed in the cation and negative ion mode. Normalized peak areas were used for quantification, and their values were log-transformed before statistical analyses. The data were pre-processed by Pareto scaling. PLS–DA, heatmap, and KEGG pathway analyses were conducted after normalizing the sample (sum normalization) and scaling the data (auto-scaling). Data were expressed as mean ± standard error of the mean (SEM). Differences were noted as significant at *p* < 0.05. The groups marked by different letters have significant differences, and the groups marked by the same letters have no significant differences.

## 3. Results

### 3.1. Effects on Body Weight and Colorectal Length

**Figure 1:**
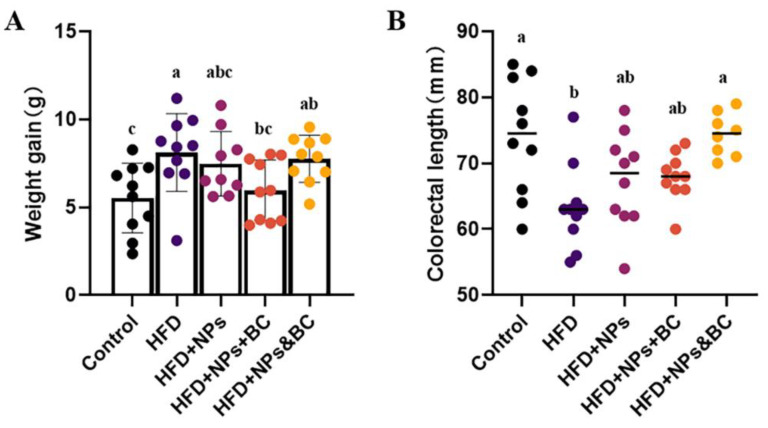
The effects on the body weight and colorectal length of mice in different groups. (**A**) The net weight gain before and after treatment in each group. (**B**) The colorectal length in different treatments. The different letters represent significant differences among groups (*p* < 0.05).

Figure 1A shows the changes in the body weight in each group. The net weight gain of the mice in the HFD group was 46.8% higher than that in the control group (*p* < 0.05), successfully establishing the high-fat-diet-induced obesity mouse model. The weight gain in the mice in the HFD + NPs + BC group was restored to a level similar to that of the control group (*p* > 0.05). However, the weight gain in the mice in the HFD + NPs&BC group was significant (an increase of 40.4%) compared to that in the control group.

Colorectal length is an important pathological indicator for analyzing colorectal inflammation. As shown in Figure 1B, the colorectal lengths of the mice in each group were also measured. Compared with the control group, the decreased colorectal lengths of the mice in the HFD group and the HFD + NPs group suggest that mice fed with a high-fat diet and TiO_2_ NPs could have reduced colorectal length. However, the colorectal lengths of the mice in the HFD + NPs&BC group were significantly more than those in the HFD group and the HFD + NPs group and recovered to the level of the control group after the mice received intervention with *B. coagulans*, demonstrating that *B. coagulans* can reduce the colorectal contraction induced by a high-fat diet and TiO_2_ NPs.

### 3.2. Effects on Inflammatory Response and Oxidative Stress

**Figure 2:**
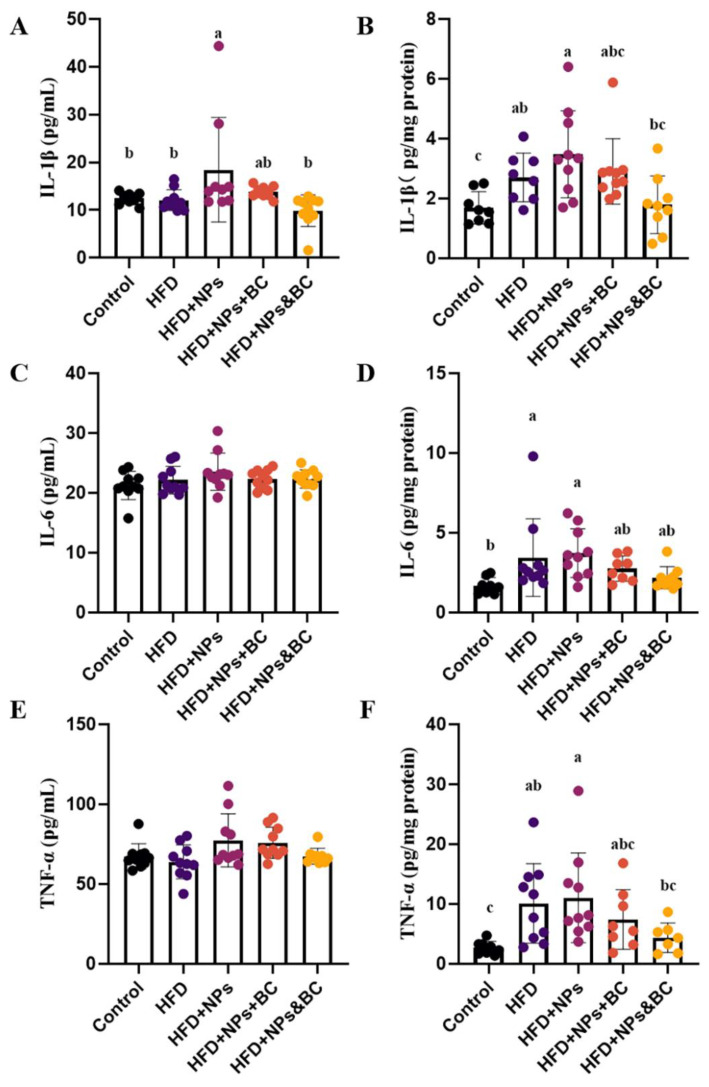
The effects of pro-inflammatory cytokine levels on serum and colon tissues in mice. (**A**) The IL-1β levels in serum, (**B**) the IL-1β levels in colon tissues, (**C**) the IL-6 levels in serum, (**D**) the IL-6 levels in colon tissues, (**E**) the TNF-α levels in serum, and (**F**) the TNF-α levels in colon tissues. The different letters represent significant differences among groups (*p* < 0.05).

As shown in Figure 2, tThe IL-1β, IL-6, and TNF-α levels were detected in serum and colon tissues (Figure 2). Our results showed that IL-1β levels in the serum significantly increased in the HFD + NPs group compared with those in the control and HFD groups, while *B. coagulans* administration significantly suppressed IL-1β production in the HFD + NPs + BC group and the HFD + NPs&BC group (*p* < 0.05) (Figure 2A). Similarly, the inhibiting effect of *B. coagulans* on IL-1β expression was observed in colon tissues (Figure 2B). Serum IL-6 levels did not differ significantly among the groups (Figure 2C), but differences in colon tissues were seen among the groups (Figure 2D). The levels of IL-6 significantly increased in the HFD group and the HFD + NPs group compared with the control group. The levels of IL-6 were, respectively, 26.0% and 41.3% lower in the HFD + NPs + BC group and the HFD + NPs&BC group than those in the HFD + NPs group. In addition, as shown in Figure 2F, the TNF-α levels in colon tissues were significantly higher in the HFD and HFD + NPs groups than in the control group. However, after *B. coagulans* treatment, the levels of TNF-α in the HFD + NPs&BC group were significantly lower than those in the HFD + NPs group (*p* < 0.05).

**Figure 3:**
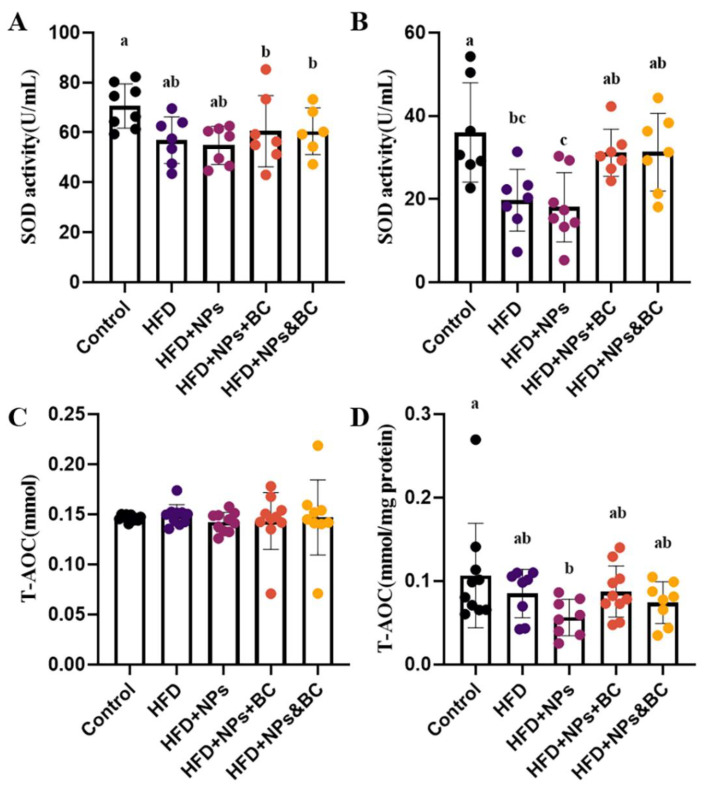
The effects on the antioxidant ability in serum and colon tissues of mice. (**A**) The SOD activities in serum, (**B**) the SOD activities in colon tissues, (**C**) the T-Aoc levels in serum, and (**D**) the T-Aoc levels in colon tissues. The different letters represent significant differences among groups (*p* < 0.05).

As a typical antioxidant metalloenzyme, SOD is usually considered to be the primary enzyme to defend against ROS-mediated damage, helping balance oxidation and antioxidant activities [24,25,26]. Our results indicated that the sensitivity of SOD in serum is lower than that in colon tissues, but the SOD activities in the HFD group and the HFD + NPs group still decrease (Figure 3A). The SOD activities in colon tissues significantly reduced in the HFD group and the HFD + NPs group compared with those in the control group, whereas *B. coagulans* intervention increased the SOD activities to a level similar to that of the control group, as shown by the SOD activities of the HFD + NPs + BC group and the HFD + NPs + BC group. These results show that the antioxidant capacity of the intestinal tract was seriously impaired by a high-fat diet and the addition of TiO_2_ NPs but the intervention with *B. coagulans* could enhance antioxidant capacity and reduce oxidative damage.

T-Aoc is one of the indicators for evaluating the total antioxidant level, reflecting the non-enzymatic antioxidant defense system [27]. As shown in Figure 3C, there was no significant difference in the serum T-Aoc levels between the groups. The T-Aoc levels in the colon tissues (Figure 3D) of the HFD + NPs group significantly decreased compared with those of the control group. However, after supplementation with *B. coagulans*, the T-Aoc levels showed only a slight upward trend, indicating the limited capacity of *B. coagulans* to inhibit the reduction of the total antioxidant level caused by TiO_2_ NPs.

### 3.3. Effects on the Diversity of Gut Microbiota

After 16S rDNA gene sequencing and quality filtering, a total of 3470 amplicon sequence variants (ASVs) were identified in the five groups. According to the classification of ASVs in each group and the identification of taxonomics (Table 2), in general, the lower the taxon and the more the ASVs seem to be detected. In the control group and the HFD group, the ASVs were relatively abundant, but they decreased significantly in the HFD + NPs group. In the HFD + NPs + BC group, the amount of ASVs had a slight rebound. However, in the HFD + NPs&BC group, the amount of ASVs increased significantly. Generally speaking, *B. coagulans* supplementation did not resist the simultaneous effects of obesity and TiO_2_ NPs but attenuated the reduction in the intestinal microbial abundance after cessation of TiO_2_ NPs intake.

**Figure 4:**
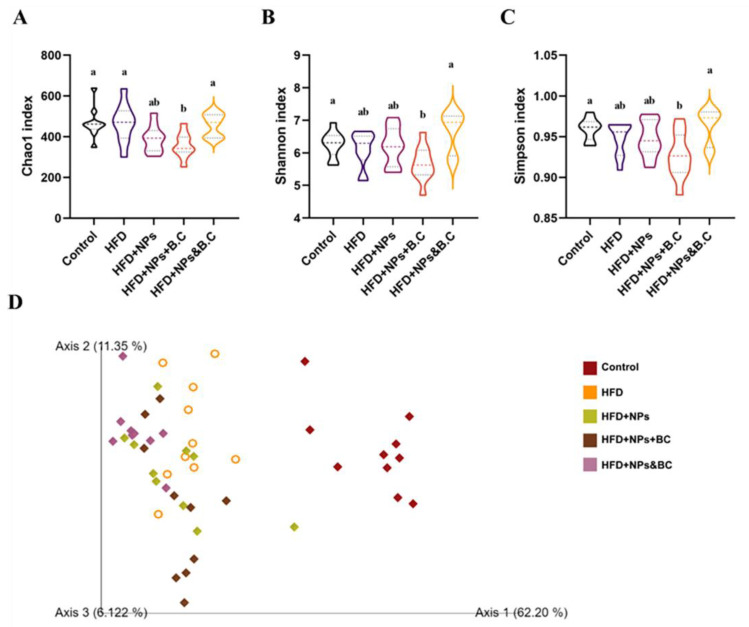
Alpha and beta diversity of gut microbiota. The (**A**) Chao1 index, (**B**) Shannon index, (**C**) Simpson index, and (**D**) PCA score plot of each group. The different letters represent significant differences among groups (*p* < 0.05).

Figure 4A–C presents the alpha diversity reflected by Chao1, Shannon, and Simpson indexes. It can be seen that the levels of alpha diversity did not differ significantly among groups, except for a slight increase in the alpha diversity in the HFD + NPs&BC group.

To further identify the effect of *B. coagulans* administration on the composition of gut microbiota in mice treated with TiO_2_ NPs, a principal component analysis (PCA) was performed. As seen in Figure 4D, there was a clear separation between the control group and other experimental groups, indicating that the gut microbiota changed greatly after the mice were fed a high-fat diet.

### 3.4. Effects on the Composition of Gut Microbiota

**Figure 5:**
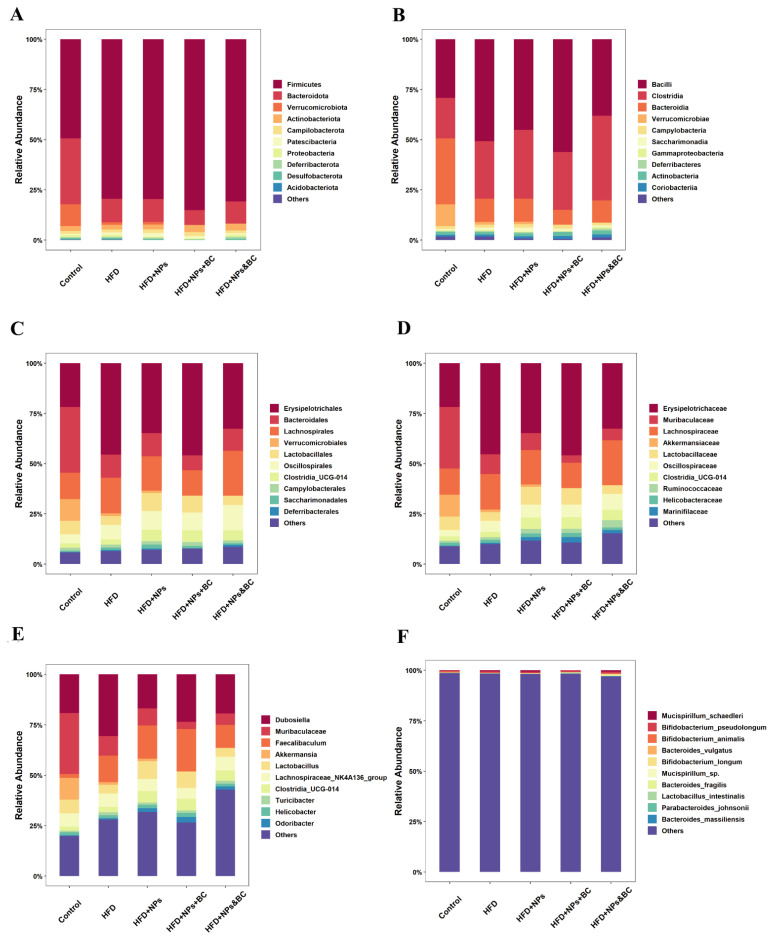
Distribution of bacterial groups at different taxonomic levels. (**A**) Phylum level, (**B**) class level, (**C**) order level, (**D**) family level, (**E**) genus level, and (**F**) species level.

Figure 5 displays the differences in the distribution of bacterial groups at different taxonomic levels. It is obvious that the lower the taxonomic level, the more prominent the regulatory effect of *B. coagulans* on intestinal microbes. To this end, we further screened and analyzed microorganisms at lower taxonomic levels.

**Figure 6:**
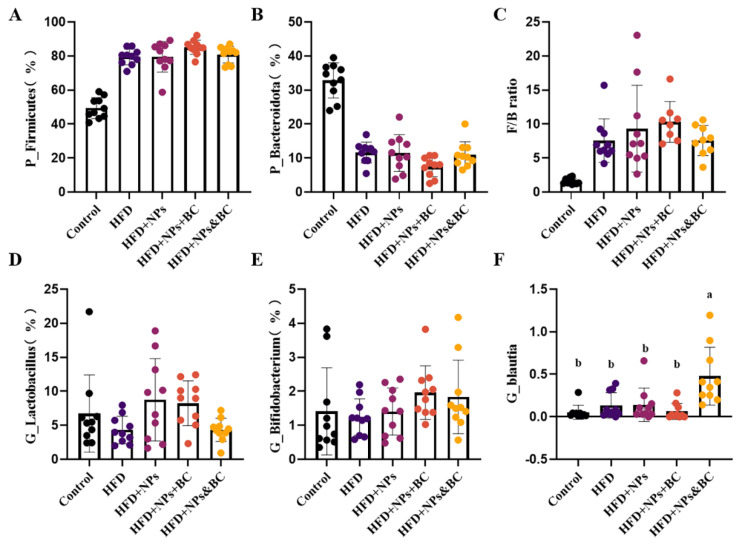
The relative abundance of microbiota at the phylum level (**A**–**C**) and the genus level (**D**–**F**). The different letters represent significant differences among groups (*p* < 0.05).

At the phylum level, the dominant intestinal bacteria in mice in the five groups were Firmicutes and Bacteroidetes. The proportion of the two dominant bacteria in the control group was approximately 82.21%, and the proportions in the HFD, HFD + NPs, HFD + NPs + BC, and HFD + NPS&BC groups were 91.12%, 91.02%, 92.54%, and 91.77%, respectively (Figure 6A–C). The proportion of the dominant bacteria Firmicutes significantly increased in the other four groups compared with the control group. The ratio of Firmicutes and Bacteroidetes (F/B) altered when the relative quantity of Firmicutes and Bacteroidetes fluctuated. Compared with the HFD + NPs group, the F/B ratio in the HFD + NPS&BC group showed a slight downward trend.

At the genus level, we screened out some characteristic bacteria (Figure 6D–F). Lactobacillus showed a downward trend after *B. coagulans* intervention, especially in the HFD + NPS&BC group. We also found a slight upward trend in the relative abundance of Bifidobacterium following *B. coagulans* intervention in the HFD + NPs + BC group and the HFD + NPs&BC group. Moreover, the relative abundance of Blautia in the HFD + NPs&BC group significantly increased, but there was no significant change in the HFD + NPs + BC group, suggesting that Blautia may be the key strain that cooperates with *B. coagulans*.

### 3.5. Effects on the Production of Short-Chain Fatty Acids

**Figure 7:**
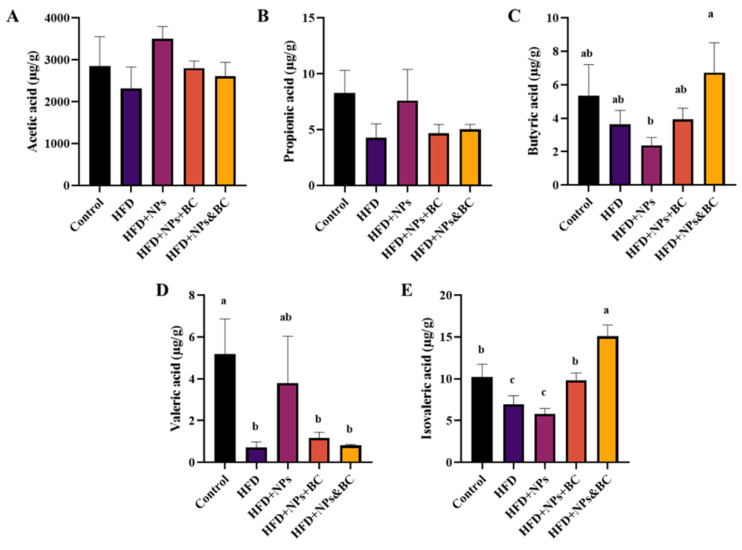
The levels of (**A**) acetic acid, (**B**) propionic acid, (**C**) butyric acid, (**D**) valeric acid, and (**E**) isovaleric acid. The different letters represent significant differences among groups (*p* < 0.05).

Short-chain fatty acids (SCFAs) are important microbial degradation metabolites and play a significant role in host health. The SCFA content in mouse feces was measured after eight weeks of different treatments. As shown in Figure 7, in the feces, there were significant differences (*p* < 0.05) in the three types of SCFAs: butyric acid, valeric acid, and isovaleric acid. Butyrate contributes a lot to colonic homeostasis and is the main energy source for colon cells [28]. In the HFD + NPs group, an inflammatory intestinal mucosa may have impaired butyrate metabolism, predisposing colon cells toward butyrate deficiency. Our results indicate that *B. coagulans* may improve cecal butyrate levels by repairing intestinal mucosal inflammation (Figure 7C). After *B. coagulans* intervention, there was a significant increase in butyrate levels in the HFD + NPs group compared to the control group. However, there were no significant changes in acetic acid and propionic acid after *B. coagulans* intervention (*p* > 0.05).

### 3.6. Effects on Fecal Metabolites

**Figure 8:**
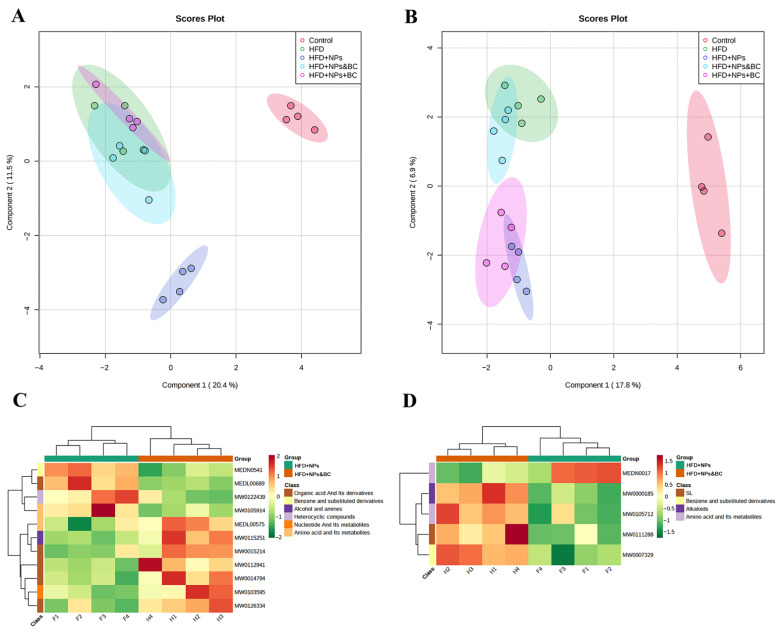
sPLS–DA score plots for each group in (**A**) the negative-ion mode and (**B**) the positive-ion mode. KEGG pathway analysis of differential metabolites for each group in (**C**) the negative-ion mode and (**D**) the positive-ion mode.

As shown in Figure 8A, the control group and the HFD + NPs group were completely separated from the other groups in the Sparse PLS discriminant analysis (sPLS–DA) score plots in the negative-ion mode. In addition, the HFD group was completely separated from the HFD + NPs group. This showed that the effect of TiO_2_ NPs on the fecal metabolites under a high-fat diet is more profound. Further analysis of the two groups subjected to *B. coagulans* intervention pointed out that there was no obvious separation between the HFD group and the HFD + NPs + BC or the HFD + NPs&BC group, indicating that *B. coagulans* could not resist the effects of a high-fat diet in mice but the damage caused by TiO_2_ NPs can be repaired by modulating fecal metabolites. Figure 8B displays the sPLS–DA scores in the positive-ion mode, which is basically consistent with the conclusions obtained in the negative-ion mode, but the distance between the HFD + NPs + BC group and the HFD + NPs group is relatively short, suggesting that the simultaneous intake of TiO_2_ NPs and *B. coagulans* has a less regulatory effect on fecal metabolites. However, the HFD + NPs&BC group remained close to the HFD group, showing that the intervention strategy of the HFD + NPs&BC group was more effective.

Furthermore, to explore the repair mechanism of *B. coagulans* in intestinal damage induced by TiO_2_ NPs, we performed a KEGG cluster analysis of differential metabolites between the HFD group and the HFD + NPs&BC group (Figure 8C,D). The KEGG metabolic pathways containing at least five differential metabolites were selected, and the relative contents of all differential metabolites in these pathways were analyzed by cluster analysis to further study the metabolite changes in the HFD + NPs group and the HFD + NPs&BC group. The results showed that, in the fecal metabolites of the HFD + NPs group, the metabolic pathways of benzene and substituted derivatives, heterocyclic compounds, and sphingolipid were up-regulated, and the metabolic pathways of amino acid and its metabolites were down-regulated. In the HFD + NPs&BC group, the metabolic pathways of organic acid and its derivatives, nucleotide and its metabolites, amino acid and its metabolites, and alkaloids were up-regulated.

## 4. Discussion

There is increasing use of TiO_2_ in daily life as a food additive and an antibacterial agent, for example, in food packaging, leading to concerns about its potential toxicity. More than 30% of the TiO_2_ used as a food additive is at the nanoscale [3]. Obese people may consume more TiO_2_ NPs than healthy people. Oral exposure of mice to TiO_2_ NPs could lead to the development and exacerbation of inflammatory bowel disease in the mice by altering their intestinal barrier function and other pathways [29]. Previous studies have pointed out that oral exposure of obese mice to TiO_2_ NPs can cause more adverse effects, such as stronger intestinal oxidative stress, increased levels of pro-inflammatory cytokines, and disturbance of intestinal microbes. However, there are few reports on intervention programs to reduce the intestinal damage caused by TiO_2_ NPs in obese individuals. Therefore, it is of great importance to explore the impact of TiO_2_ NPs on intestinal health from multiple perspectives and methods to alleviate this negative impact. Here, *B. coagulans* used in two intervention methods was investigated for its repair effect on TiO_2_-NP-induced damage in mice fed a high-fat diet.

According to previous research, we established an obese mouse model and added 0.2% TiO_2_ NPs to the high-fat diet to simulate the addition of TiO_2_ NPs as a food additive in a daily high-fat diet. The results of mice weight showed that the simultaneous action of *B. coagulans* and TiO_2_ NPs had a more positive regulatory effect on the body weight of mice. In addition, the colorectal lengths suggested that *B. coagulans* can prevent colorectal contraction caused by a high-fat diet and TiO_2_ NPs. However, the results of blood glucose and blood lipids exhibited that *B. coagulans* did not reduce the levels of fasting blood glucose and blood lipids in mice (Figure S1). Therefore, we preliminarily concluded that the administration of *B. coagulans* can restore the body weight and colorectal length of mice to the normal level but does not address the imbalance of glucose and lipid metabolism caused by obesity.

When TiO_2_ NPs pass through the digestive system and finally reach the intestine, they may enrich the inflammatory cells in the intestinal mucosa, which secrete a large number of inflammatory mediators, cytokines, toxins, and oxygen-free radicals [30]. Cytokines play an important role in the intestinal immune system [31,32]. As a part of the intestinal tract, the intestinal immune system can regulate the intestinal barrier function [33,34]. It is reported that TiO_2_ NPs can aggravate intestinal inflammation by activating innate and adaptive immune responses [35]. Pro-inflammatory cytokines, such as IL-1β, IL-6, and TNF-α, serve to stimulate inflammatory responses and promote the occurrence of intestinal diseases [36,37]. In this study, the increased levels of IL-1β, IL-6, and TNF-α in colon tissues in the HFD group and the HFD + NPs group indicate the higher inflammation levels resulting from the exposure to TiO_2_ NPs and the high-fat diet, which is consistent with previous studies [38,39,40,41,42]. However, *B. coagulans* intervention significantly reduced the inflammatory response, as shown by the reduced levels of pro-inflammatory cytokines in the HFD + NPs + BC group and the HFD + NPs&BC group. Inflammation and oxidative stress are closely related to pathophysiological processes, and one can be easily provoked by the other [43]. Inflammation is usually the initial response to tissue damage, with conditions leading to tissue damage that may be promoted by oxidative stress [44,45,46,47]. In fact, oxidative stress results from an imbalance between the production of pro-oxidants and their neutralization by antioxidants [48]. Here, the antioxidant ability was further evaluated, where the elevated SOD activities in colon tissues in the HFD + NPs + BC group and the HFD + NPs&BC group reaffirmed the protective effect of *B. coagulans* against intestinal damage. Moreover, the greater fluctuation of pro-inflammatory cytokines and oxidative stress levels in colon tissues t could be because intestinal tissue is more sensitive to inflammation than serum.

In addition, TiO_2_ NPs have a certain antibacterial activity [49,50] and can promote microbial dysregulation [51], so gut microbes may be highly sensitive to them. Thus, we subsequently studied the changes in gut microbes. We found that *B. coagulans* could also ameliorate the reduction in intestinal microbial species resulting from exposure to TiO_2_ NPs (Table 2) and modulate the composition of gut microbiota (Figure 5 and Figure 6). Previously, our research group found that the relative abundance of Lactobacillus in the guts of mice exposed to TiO_2_ NPs increased in a dose-dependent manner [10], which corresponded to other research, and the researchers suggested that the increase in Lactobacillus may be a key factor in the influence of TiO_2_ NPs on gut microbiota [35]. Lactobacillus_gasseri was able to cause gangrene, leading to increased ROS production [52]. Notably, a reduction in Lactobacillus was observed in *B. coagulans* treatment. Blautia has been reported as a potential probiotic with beneficial metabolic activity for host health, protecting against inflammation and promoting SCFA activity [53]. The significant increase in the levels of Blautia in the HFD + NPs&BC group also supported the beneficial effect of *B. coagulans*. At the genus level, in addition to the top ten abundant microorganisms, we summed up the relative abundances of the remaining microorganisms (Figure S2). The significantly increased abundance of the HFD + NPs&BC group may be the reason for the previous increase in alpha diversity in this group.

Alterations in the gut microbiota may deliver signals through the gut and generate bacterial metabolites that change metabolism at different levels [35]. We performed fecal metabolomic analysis to understand the metabolism of gut microbes. Metabolites obtained in different modes were visualized using sPLS–DA. The results showed that the effectiveness of *B. coagulans* was influenced by the intervention methods and that the administration of TiO_2_ NPs followed by *B. coagulans* (HFD + NPs&BC group) was more effective than the administration of TiO_2_ NPs and *B. coagulans* simultaneously (HFD + NPs + BC group). In other words, for individuals whose intestinal health has already been impaired by TiO_2_ NPs, it is more effective to use *B. coagulans* after stopping their exposure to TiO_2_ NPs. To further explore the reasons for the better intervention effect in the HFD + NPs&BC group, we clustered the differential metabolites (Tables S4 and S5) between HFD + NPs and HFD + NPs&BC groups to further study the metabolic pathways. The metabolic pathways showed significant differences. Sphingolipid pathways were up-regulated, and the metabolic pathway of amino acid and its metabolites was down-regulated in the HFD + NPs group. However, amino acid and its metabolites were up-regulated in the HFD + NPs&BC group. Sphingolipid plays an important role in gastrointestinal immune homeostasis. Metabolites in this pathway are crucial in inflammatory signaling pathways, and some related metabolites can affect the integrity and function of the intestinal barrier and promote inflammation [54]. The amino acid metabolism pathway is the premise of various metabolic pathways, and amino acid metabolism disorders are related to the occurrence of diseases such as liver cirrhosis [55]. It can be seen that *B. coagulans* plays an important role in restoring the homeostasis of sphingolipids and amino acid metabolism.

According to our results, TiO_2_ NPs affected gut health in mice on a high-fat diet, leading to, for example, oxidative stress, inflammatory responses, and disorders of gut microbiota and gut-associated metabolism. However, dietary intervention with probiotics was found to attenuate intestinal damage. *B. coagulans* could alleviate the intestinal inflammation and oxidative stress caused by TiO_2_ NPs. In addition, the beneficial effects of *B. coagulans* were manifested as the modulation of gut microbiota and gut-associated metabolism. The regulation of gut microbiota was represented by decreased Lactobacillus and increased Bifidobacterium and Blautia. The opsonization of metabolic disorders was shown by the homeostasis of sphingolipids and amino acid metabolism. Notably, compared with the simultaneous intervention of TiO_2_ NPs and *B. coagulans*, *B. coagulans* intervention following the exposure to TiO_2_ NPs is more effective.

## 5. Conclusions

In this study, we focused on seeking preventive or therapeutic intervention programs to counteract the negative effects of TiO_2_ NPs on the gut. Our results illustrated that *B. coagulans* could mitigate intestinal damage by decreasing the inflammatory response and oxidative stress levels and modulating intestinal microbial community structure and its metabolic profile, leading to a healthy tendency. Importantly, the current study pointed out that the intervention method used influenced *B. coagulans* effectiveness, as the administration of TiO_2_ NPs followed by *B. coagulans* displayed a greater protective effect against intestinal injury. This research highlights the importance of dietary interventions in preventing or reducing the intestinal burden after dietary exposure to TiO_2_ NPs, and more intervention modalities to restore intestinal health need to be investigated.

## Figures and Tables

**Table 1:** Animal groups and treatments.

Groups, *n* = 10	Treatments *	Duration
Control	Normal diet	8 weeks
HFD	High-fat diet	8 weeks
HFD + NPs	High-fat diet + TiO_2_ NPs	8 weeks
HFD + NPs + BC	High-fat diet + TiO_2_ NPs + *B. coagulans*	8 weeks
HFD + NPs&BC	High-fat diet + TiO_2_ NPs, followed by a high-fat diet + *B. coagulans*	High-fat diet + TiO_2_ NPs for 4 weeks, followed by a high-fat diet + *B. coagulans* MTCC 5856 for 4 weeks

* The daily dose was equivalent to 0.2% of the body weight of the mice for TiO_2_ NPs and 10^9^ CFUs for *B. coagulans* MTCC 5856.

**Table 2:** Classification of ASVs in each group and the identification of taxonomics.

Group	Domain	Phylum	Class	Order	Family	Genus	Species
Control	1	22	60	123	171	244	93
HFD	1	23	54	117	173	275	111
HFD + NPs	1	13	17	37	55	138	81
HFD + NPs + BC	1	13	19	41	56	145	80
HFD + NPs&BC	1	13	18	43	72	163	109

## Data Availability

The data presented in this study are available in this article.

## Supplementary Materials

The following supporting information can be downloaded at: https://www.mdpi.com/article/10.3390/foods11213368/s1. Table S1: Key parameters for GC-MS analysis; Table S2: The chromatographic column and mobile phase gradient program; Table S3: Mass spectrometry conditions in negative ion and positive ion modes; Table S4: Differential me-tabolites in the negative ion mode and its classification; Table S5: Differential metabolites in the positive ion mode and its classification; Figure S1: The levels of blood glucose, triglyceride and total cholesterol in different groups; Figure S2: The sum of the other microorganisms at the genus level.

## References

[1] Baranowska-Wójcik E., Szwajgier D., Winiarska-Mieczan A. (2022). A review of research on the impact of E171/TiO_2_ NPs on the digestive tract. J. Trace Elements Med. Biol..

[2] Baranowska-Wójcik E. (2021). Factors Conditioning the Potential Effects TiO_2_ NPs Exposure on Human Microbiota: A Mini-Review. Biol. Trace Element Res..

[3] Weir A., Westerhoff P., Fabricius L., Hristovski K., von Goetz N. (2012). Titanium Dioxide Nanoparticles in Food and Personal Care Products. Environ. Sci. Technol..

[4] McClements D.J., Xiao H., Demokritou P. (2017). Physicochemical and colloidal aspects of food matrix effects on gastrointestinal fate of ingested inorganic nanoparticles. Adv. Colloid Interface Sci..

[5] Heringa M.B., Geraets L., Van Eijkeren J.C.H., Vandebriel R.J., De Jong W.H., Oomen A.G. (2016). Risk assessment of titanium dioxide nanoparticles via oral exposure, including toxicokinetic considerations. Nanotoxicology.

[6] Younes M., Aquilina G., Castle L., Engel K.-H., Fowler P., Fernandez M.J.F., Fürst P., Gundert-Remy U., Gürtler R., Husoy T. (2021). Safety assessment of titanium dioxide (E171) as a food additive. EFSA J..

[7] Limage R., Tako E., Kolba N., Guo Z., García-Rodríguez A., Marques C.N.H., Mahler G.J. (2020). TiO_2_ Nanoparticles and Commensal Bacteria Alter Mucus Layer Thickness and Composition in a Gastrointestinal Tract Model. Small.

[8] Powell J.J., Ainley C.C., Harvey R.S.J., Mason I.M., Kendall M.D., Sankey E.A., Dhillon A.P.H., Thompson R.P. (1996). Characterisation of inorganic microparticles in pigment cells of human gut associated lymphoid tissue. Gut.

[9] Brun E., Barreau F., Veronesi G., Fayard B., Sorieul S., Chanéac C., Carapito C., Rabilloud T., Mabondzo A., Herlin-Boime N. (2014). Titanium dioxide nanoparticle impact and translocation through ex vivo, in vivo and in vitro gut epithelia. Part. Fibre Toxicol..

[10] Yang C., Tan Y., Li F., Wang H., Lin Y., Lu F., Zhao H. (2022). Intestinal Microecology of Mice Exposed to TiO_2_ Nanoparticles and Bisphenol A. Foods.

[11] Hassanein K.M.A., El-Amir Y.O. (2017). Protective effects of thymoquinone and avenanthramides on titanium dioxide nanoparticles induced toxicity in Sprague-Dawley rats. Pathol.-Res. Pract..

[12] Jafari A., Rasmi Y., Hajaghazadeh M., Karimipour M. (2018). Hepatoprotective effect of thymol against subchronic toxicity of titanium dioxide nanoparticles: Biochemical and histological evidences. Environ. Toxicol. Pharmacol..

[13] Ruiz P.A., Morón B., Becker H.M., Lang S., Atrott K., Spalinger M.R., Scharl M., Wojtal K.A., Fischbeck-Terhalle A., Frey-Wagner I. (2017). Titanium dioxide nanoparticles exacerbate DSS-induced colitis: Role of the NLRP3 inflammasome. Gut.

[14] Cao X., Han Y., Gu M., Du H., Song M., Zhu X., Ma G., Pan C., Wang W., Zhao E. (2020). Foodborne Titanium Dioxide Nanoparticles Induce Stronger Adverse Effects in Obese Mice than Non-Obese Mice: Gut Microbiota Dysbiosis, Colonic Inflammation, and Proteome Alterations. Small.

[15] Sonnenburg J.L., Bäckhed F. (2016). Diet–microbiota interactions as moderators of human metabolism. Nature.

[16] Natividad J.M., Lamas B., Pham H.P., Michel M.-L., Rainteau D., Bridonneau C., da Costa G., Van Hylckama Vlieg J., Sovran B., Chamignon C. (2018). Bilophila wadsworthia aggravates high fat diet induced metabolic dysfunctions in mice. Nat. Commun..

[17] Konuray G., Erginkaya Z. (2018). Potential Use of Bacillus coagulans in the Food Industry. Foods.

[18] Majeed M., Majeed S., Arumugam S., Ali F., Beede K. (2020). Comparative evaluation for thermostability and gastrointestinal survival of probiotic *Bacillus coagulans* MTCC 5856. Biosci. Biotechnol. Biochem..

[19] Shinde T., Perera A.P., Vemuri R., Gondalia S.V., Karpe A.V., Beale D.J., Shastri S., Southam B., Eri R., Stanley R. (2019). Synbiotic Supplementation Containing Whole Plant Sugar Cane Fibre and Probiotic Spores Potentiates Protective Synergistic Effects in Mouse Model of IBD. Nutrients.

[20] Majeed M., Nagabhushanam K., Arumugam S., Majeed S., Ali F. (2018). Bacillus coagulans MTCC 5856 for the management of major depression with irritable bowel syndrome: A randomised, double-blind, placebo controlled, multi-centre, pilot clinical study. Food Nutr. Res..

[21] Majeed M., Nagabhushanam K., Natarajan S., Sivakumar A., Ali F., Pande A., Majeed S., Karri S.K. (2016). Bacillus coagulans MTCC 5856 supplementation in the management of diarrhea predominant Irritable Bowel Syndrome: A double blind ran-domized placebo controlled pilot clinical study. Nutr. J..

[22] Majeed M., Nagabhushanam K., Natarajan S., Arumugam S., Pande A., Majeed S., Ali F. (2016). A Double-Blind, Place-bo-Controlled, Parallel Study Evaluating the Safety of Bacillus coagulans MTCC 5856 in Healthy Individuals. J. Clin. Toxicol..

[23] Majeed M., Nagabhushanam K., Natarajan S., Sivakumar A., Ruiter T.E.-d., Booij-Veurink J., de Vries Y.P., Ali F. (2016). Eval-uation of genetic and phenotypic consistency of Bacillus coagulans MTCC 5856: A commercial probiotic strain. World J. Microbiol. Biotechnol..

[24] Wang C., Nie G., Yang F., Chen J., Zhuang Y., Dai X., Liao Z., Yang Z., Cao H., Xing C. (2019). Molybdenum and cadmium co-induce oxidative stress and apoptosis through mitochondria-mediated pathway in duck renal tubular epithelial cells. J. Hazard. Mater..

[25] Pandey S., Parvez S., Sayeed I., Haque R., Bin-Hafeez B., Raisuddin S. (2003). Biomarkers of oxidative stress: A comparative study of river Yamuna fish Wallago attu (Bl. & Schn.). Sci. Total Environ..

[26] Gao M., Liu Y., Song Z. (2019). Effects of polyethylene microplastic on the phytotoxicity of di-n-butyl phthalate in lettuce (*Lactuca sativa* L. var. ramosa Hort). Chemosphere.

[27] Momeni H.R., Eskandari N. (2017). Effect of curcumin on kidney histopathological changes, lipid peroxidation and total antioxidant capacity of serum in sodium arsenite-treated mice. Exp. Toxicol. Pathol..

[28] Mrozinska S., Kapusta P., Gosiewski T., Sroka-Oleksiak A., Ludwig-Słomczyńska A.H., Matejko B., Kiec-Wilk B., Bulanda M., Malecki M.T., Wolkow P.P. (2021). The Gut Microbiota Profile According to Glycemic Control in Type 1 Diabetes Patients Treated with Personal Insulin Pumps. Microorganisms.

[29] Barreau F., Tisseyre C., Ménard S., Ferrand A., Carriere M. (2021). Titanium dioxide particles from the diet: Involvement in the genesis of inflammatory bowel diseases and colorectal cancer. Part. Fibre Toxicol..

[30] Liang Y., Li C., Liu B., Zhang Q., Yuan X., Zhang Y., Ling J., Zhao L. (2019). Protective effect of extracorporeal membrane oxygenation on intestinal mucosal injury after cardiopulmonary resuscitation in pigs. Exp. Ther. Med..

[31] Polinska B., Matowicka-Karna J., Kemona H. (2009). The cytokines in inflammatory bowel disease. Postep. Hig. I Med. Dosw..

[32] Moldoveanu A.C., Diculescu M., Braticevici C.F. (2015). Cytokines in inflammatory bowel disease. Rom. J. Intern. Med. = Rev. Roum. De Med. Interne.

[33] Xu C.-L., Sun R., Qiao X.-J., Xu C.-C., Shang X.-Y., Niu W.-N. (2014). Protective effect of glutamine on intestinal injury and bacterial community in rats exposed to hypobaric hypoxia environment. World J. Gastroenterol..

[34] Gong Y., Li H., Li Y. (2016). Effects of *Bacillus subtilis* on Epithelial Tight Junctions of Mice with Inflammatory Bowel Disease. J. Interf. Cytokine Res..

[35] Chen Z., Han S., Zhou D., Zhou S., Jia G. (2019). Effects of oral exposure to titanium dioxide nanoparticles on gut microbiota and gut-associated metabolism *in vivo*. Nanoscale.

[36] Moldoveanu B., Otmishi P., Jani P., Walker J., Sarmiento X., Guardiola J., Saad M., Yu J. (2009). Inflammatory mechanisms in the lung. J. Inflamm. Res..

[37] Abbasi-Oshaghi E., Mirzaei F., Pourjafar M. (2019). NLRP3 inflammasome, oxidative stress, and apoptosis induced in the intestine and liver of rats treated with titanium dioxide nanoparticles: In vivo and in vitro study. Int. J. Nanomed..

[38] Mu W., Wang Y., Huang C., Fu Y., Li J., Wang H., Jia X., Ba Q. (2019). Effect of Long-Term Intake of Dietary Titanium Dioxide Nanoparticles on Intestine Inflammation in Mice. J. Agric. Food Chem..

[39] Cui Y., Liu H., Zhou M., Duan Y., Li N., Gong X., Hu R., Hong M., Hong F. (2011). Signaling pathway of inflammatory responses in the mouse liver caused by TiO_2_ nanoparticles. J. Biomed. Mater. Res. Part A.

[40] Kongseng S., Yoovathaworn K., Wongprasert K., Chunhabundit R., Sukwong P., Pissuwan D. (2016). Cytotoxic and inflammatory responses of TiO_2_ nanoparticles on human peripheral blood mononuclear cells. J. Appl. Toxicol..

[41] Duan Y., Zeng L., Zheng C., Song B., Li F., Kong X., Xu K. (2018). Inflammatory Links Between High Fat Diets and Diseases. Front. Immunol..

[42] Cani P.D., Bibiloni R., Knauf C., Waget A., Neyrinck A.M., Delzenne N.M., Burcelin R. (2008). Changes in Gut Microbiota Control Metabolic Endotoxemia-Induced Inflammation in High-Fat Diet-Induced Obesity and Diabetes in Mice. Diabetes.

[43] Shi Q., Tang J., Wang L., Liu R., Giesy J.P. (2021). Combined cytotoxicity of polystyrene nanoplastics and phthalate esters on human lung epithelial A549 cells and its mechanism. Ecotoxicol. Environ. Saf..

[44] Thomson A., Hemphill D., Jeejeebhoy K. (1998). Oxidative Stress and Antioxidants in Intestinal Disease. Dig. Dis..

[45] Liu P., Wang Y., Yang G., Zhang Q., Meng L., Xin Y., Jiang X. (2021). The role of short-chain fatty acids in intestinal barrier function, inflammation, oxidative stress, and colonic carcinogenesis. Pharmacol. Res..

[46] Qiao R., Sheng C., Lu Y., Zhang Y., Ren H., Lemos B. (2019). Microplastics induce intestinal inflammation, oxidative stress, and disorders of metabolome and microbiome in zebrafish. Sci. Total Environ..

[47] Bondia-Pons I., Ryan L., Martinez J.A. (2012). Oxidative stress and inflammation interactions in human obesity. J. Physiol. Biochem..

[48] Ahamed M., Akhtar M.J., Khan M.M., Alrokayan S., Alhadlaq H. (2019). Oxidative stress mediated cytotoxicity and apoptosis response of bismuth oxide (Bi_2_O_3_) nanoparticles in human breast cancer (MCF-7) cells. Chemosphere.

[49] Sohm B., Immel F., Bauda P., Pagnout C. (2015). Insight into the primary mode of action of TiO_2_ nanoparticles on *Escherichia coli* in the dark. Proteomics.

[50] Kong H., Song J., Jang J. (2010). Photocatalytic Antibacterial Capabilities of TiO_2_−Biocidal Polymer Nanocomposites Synthesized by a Surface-Initiated Photopolymerization. Environ. Sci. Technol..

[51] Schwarzfischer M., Rogler G. (2022). The Intestinal Barrier—Shielding the Body from Nano- and Microparticles in Our Diet. Metabolites.

[52] Tleyjeh I.M., Routh J., Qutub M.O., Lischer G., Liang K.V., Baddour L.M. (2004). Lactobacillus gasseri causing Fournier’s gangrene. Scand. J. Infect. Dis..

[53] Liu X., Mao B., Gu J., Wu J., Cui S., Wang G., Zhao J., Zhang H., Chen W. (2021). *Blautia*—A new functional genus with potential probiotic properties?. Gut Microbes.

[54] Rohrhofer J., Zwirzitz B., Selberherr E., Untersmayr E. (2021). The Impact of Dietary Sphingolipids on Intestinal Microbiota and Gastrointestinal Immune Homeostasis. Front. Immunol..

[55] Wei X., Jiang S., Zhao X., Li H., Lin W., Li B., Lu J., Sun Y., Yuan J. (2016). Community-Metabolome Correlations of Gut Microbiota from Child-Turcotte-Pugh of A and B Patients. Front. Microbiol..

