# Supplementary material for: Bacillus coagulans Alleviates Intestinal Damage Induced by TiO2 Nanoparticles in Mice on a High-Fat Diet
Source: Foods. 2022 Oct 26;11(21):3368. doi: 10.3390/foods11213368 (PMC9655532; doi:10.3390/foods11213368)
Supplement: Supplementary file 1 [file foods-11-03368-s001.zip › foods-1939882-supplementary.pdf]

Supplementary Information for

Bacillus Coagulans alleviates intestinal damage induced by TiO_2 nanoparticles in high-fat diet mice

Qingying Shi [1], Chen Yang [1], Bingjie Zhang [1], Dongxiao Chen [1], Hongbin Wang [1,2], Fuping Lu [1,2], Huabing Zhao [1,2,*]

[1] College of Biotechnology, Tianjin University of Science and Technology, 9 TEDA 13th Street, Tianjin 300457, China

[2] Key Laboratory of Industrial Fermentation Microbiology, Ministry of Education, Tianjin University of Science and Technology, Tianjin 300450, China

[*] Correspondence: zhaohuabing@tust.edu.cn

Table S1 Key parameters for GC-MS analysis.

Items	Parameters
Injection Volume	1μL
Front Inlet Mode	No spilt
Carrier Gas	Helium
Column	HP-5 MS (60m x 0.32mm x 0.25um)
Column Flow	1 mL/min
Oven Temperature Ramp	50 ℃ hold on 2min, raised to 250 ℃ at a rate of 10 ℃/min, hold on 15 min, raised to 320 ℃ at a rate of 10 ℃/min, hold on 10 min
Front Injection Temperature	250℃
Ion Source Temperature	230℃
Ionization Mode	EI
Electron Energy	70 eV
Scan range	50-700 m/z

Table S2 The chromatographic column and mobile phase gradient program.

Time（min）	Flow（mL/min）	A（%）	B（%）
0.0	0.40	95	5
11.0	0.40	10	90
12.0	0.40	10	90
12.1	0.40	95	5
14.0	0.40	95	5

Table S3 Mass spectrometry conditions in negative ion and positive ion modes.

Items	ESI+	ESI-
Ion Source Voltage (V)	2500	1500
Aux Gas Flow Rate (L/min)	8	8
Fragmentor (V)	135	135
Ion Source Temperature (℃)	325	325
Shealth Gas Temperature (℃)	15	15
Shealth Gas Flow Rate (L/min)	35	35

Table S4 Differential metabolites in the negative ion mode and its classification.

Compounds	Class	p_value	FC	Log2FC	Type
2-Methoxyestradiol	Alcohol and amines	0.042	0.206	-2.276	down
Salidroside	Alcohol and amines	0.016	2.265	1.179	up
Enterolactone	Aldehyde,Ketones,Esters	0.019	2.579	1.367	up
Deoxysappanone B 7,3'-dimethyl ether	Aldehyde,Ketones,Esters	0.021	2.292	1.197	up
11-Dihydro-12-norneoquassin	Aldehyde,Ketones,Esters	0.011	0.455	-1.136	down
(E)-2-Butyl-2-octenal	Aldehyde,Ketones,Esters	0.000	2.387	1.255	up
L-Isoleucine	Amino acid and Its metabolites	0.035	2.112	1.079	up
N5-(1-Iminoethyl)-L-ornithine	Amino acid and Its metabolites	0.038	2.189	1.130	up
Aspartic acid	Amino acid and Its metabolites	0.035	0.273	-1.875	down
Ile-Lys-Ala-Arg	Amino acid and Its metabolites	0.033	2.111	1.078	up
Arg-Ala-Leu-Lys	Amino acid and Its metabolites	0.033	2.737	1.453	up
(6,7-Dimethoxy-1,4-dihydroindeno[1,2-c]pyrazol-3-yl)(3-fluorophenyl)amine	Benzene and substituted derivatives	0.009	0.362	-1.465	down
VULPINIC ACID	Benzene and substituted derivatives	0.009	2.554	1.353	up
1-O-Cinnamoylglucose	Benzene and substituted derivatives	0.008	2.438	1.285	up
Phenol,3-[4-(4-morpholinyl)pyrido[3',2'	Benzene and substituted derivatives	0.007	3.060	1.614	up
DL-3,4-Dihydroxyphenyl glycol	Benzene and substituted derivatives	0.002	0.421	-1.247	down
Halofenozide	Benzene and substituted derivatives	0.009	2.539	1.344	up
Carbendazim	Benzene and substituted derivatives	0.017	0.413	-1.274	down
3-[4-(2-methylbutan-2-yl)phenoxy]benzoic acid	Benzene and substituted derivatives	0.033	0.169	-2.568	down
25-Hydroxyvitamin D3-26,23-lactone	CoEnzyme and vitamins	0.047	2.031	1.022	up
Resolvin E1	FA	0.044	0.354	-1.497	down
1-Mar	FA	0.008	2.690	1.428	up
7-Chloro-L-tryptophan	Heterocyclic compounds	0.028	0.280	-1.838	down

Furmecyclox	Heterocyclic compounds	0.017	2.303	1.204	up
Equol	Heterocyclic compounds	0.000	3.222	1.688	up
Ubiquinone-4	Heterocyclic compounds	0.038	2.949	1.560	up
Riluzole	Heterocyclic compounds	0.035	0.435	-1.202	down
Flunitrazepam	Heterocyclic compounds	0.000	2.113	1.079	up
Riesling acetal	Heterocyclic compounds	0.033	2.229	1.156	up
(Z)-7-Hexadecen-1,16-olide	Heterocyclic compounds	0.030	2.013	1.009	up
8-iso PGF3	Hormones and hormone related compounds	0.031	0.311	-1.687	down
Inosine	Nucleotide And Its metabolites	0.033	3.598	1.847	up
Glycochenodeoxycholic acid	Organic acid And Its derivatives	0.003	0.290	-1.788	down
Pyridoxal phosphate	Organic acid And Its derivatives	0.016	2.273	1.184	up
8-Amino-7-oxononanoic acid	Organic acid And Its derivatives	0.026	2.063	1.045	up
Hydroxyphenyllactic acid	Organic acid And Its derivatives	0.037	0.215	-2.220	down
2-[4-[2-[4-cyclohexylbutyl(cyclohexylcarbamoyl)amino]ethyl]phenyl]sulfanyl-2-methylpropanoic acid	Organic acid And Its derivatives	0.045	2.412	1.270	up
Phenylpyruvic Acid	Organic acid And Its derivatives	0.006	0.418	-1.258	down
Vanillic Acid	Organic acid And Its derivatives	0.039	0.326	-1.617	down
5-Hydroxyindole-3-acetic acid	Organic acid And Its derivatives	0.019	3.116	1.640	up
4-(2-Aminophenyl)-2,4-dioxobutanoic acid	Organic acid And Its derivatives	0.023	2.463	1.301	up
Melilotocarpan C	Others	0.008	2.536	1.342	up
Thiosulfate	Others	0.001	3.456	1.789	up
Docosahexaenoyl Serotonin	Tryptamines,Cholines,Pigments	0.049	2.057	1.041	up
Phenylpyruvic Acid	Organic acid And Its derivatives	0.042	0.206	-2.276	down
Vanillic Acid	Organic acid And Its derivatives	0.016	2.265	1.179	down

5-Hydroxyindole-3-acetic acid	Organic acid And Its derivatives	0.019	2.579	1.367	up
4-(2-Aminophenyl)-2,4-dioxobutanoic acid	Organic acid And Its derivatives	0.021	2.292	1.197	up

Table S5 Differential metabolites in the positive ion mode and its classification.

Compounds	Class	p_value	FC	Log2FC	Type
n-Oleoylethanolamine	Alcohol and amines	0.036	2.600	1.378	up
(E)-2-aminooctadec-4-ene-1,3-diol	Alcohol and amines	0.042	2.409	1.268	up
Retinyl propionate	Aldehyde,Ketones,Esters	0.020	2.056	1.040	up
Germacrone	Aldehyde,Ketones,Esters	0.001	2.546	1.348	up
Donepezil	Aldehyde,Ketones,Esters	0.013	2.067	1.047	up
beta-Sitostenone	Aldehyde,Ketones,Esters	0.004	2.322	1.215	up
Codeine	Alkaloids	0.000	2.274	1.185	up
Methylergonovine	Alkaloids	0.043	2.457	1.297	up
L-Pyroglutamic Acid	Amino acid and Its metabolites	0.044	0.478	-1.064	down
Leu-Leu-Phe	Amino acid and Its metabolites	0.002	2.075	1.053	up
Ile-Met	Amino acid and Its metabolites	0.047	0.362	-1.465	down
Phe-Val	Amino acid and Its metabolites	0.022	2.694	1.430	up
Argininosuccinic acid	Amino acid and Its metabolites	0.018	2.484	1.313	up
1-amino-1-cyclobutane carboxylic acid	Amino acid and Its metabolites	0.035	2.282	1.190	up
Leu-Leu-Glu	Amino acid and Its metabolites	0.011	0.456	-1.132	down
Pro-Tyr	Amino acid and Its metabolites	0.017	0.271	-1.884	down
L-Homoarginine	Amino acid and Its metabolites	0.006	2.192	1.132	up
Leu-Asn	Amino acid and Its metabolites	0.006	0.366	-1.450	down
2-Octanamidoacetic acid	Amino acid and Its metabolites	0.005	2.215	1.147	up
N-Acetyl-L-Leucine	Amino acid and Its metabolites	0.005	0.464	-1.109	down

Tyr-Ser-Leu	Amino acid and Its metabolites	0.024	9.319	3.220	up
Tyr-His-Val	Amino acid and Its metabolites	0.036	2.398	1.262	up
Lys-Asn-Ile	Amino acid and Its metabolites	0.000	2.339	1.226	up
Leu-Ala-Tyr	Amino acid and Its metabolites	0.007	3.499	1.807	up
Ile-Asn-Lys	Amino acid and Its metabolites	0.002	2.683	1.424	up
Salmeterol	Benzene and substituted derivatives	0.019	0.455	-1.136	down
1,2,3-Benzenetriol	Benzene and substituted derivatives	0.032	0.370	-1.432	down
Formoterol	Benzene and substituted derivatives	0.007	43.960	5.458	up
Homovanillin	Benzene and substituted derivatives	0.002	2.851	1.511	up
Mescaline	Benzene and substituted derivatives	0.032	2.080	1.057	up
Oxeladin	Benzene and substituted derivatives	0.020	2.854	1.513	up
Carbetapentane	Benzene and substituted derivatives	0.027	2.419	1.274	up
5-benzyl-5-(pyridin-3-yl)imidazolidine-2,4-dione	Benzene and substituted derivatives	0.008	2.497	1.320	up
15-Lipoxygenase Inhibitor I	Heterocyclic compounds	0.004	0.449	-1.156	down
Callystatin A	Heterocyclic compounds	0.007	117.984	6.882	up
Cyanazine	Heterocyclic compounds	0.043	0.436	-1.197	down
Pregnandiol	Hormones and hormone related compounds	0.040	2.092	1.065	up
N-hydroxy-2-(4-hydroxyphenyl)ethanimine oxide	Others	0.032	2.005	1.004	up
Sphingosine	SL	0.012	2.108	1.076	up

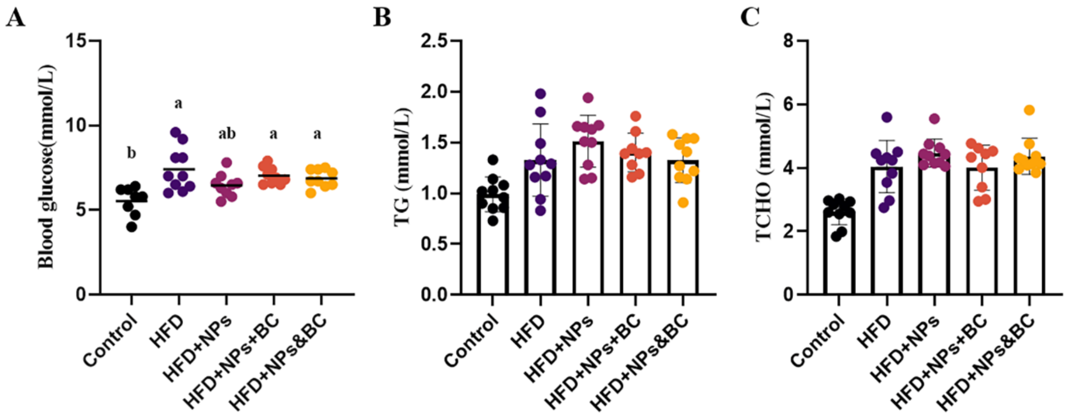

Figure S1 The levels of (A) Blood glucose; (B) Triglyceride and (C) Total cholesterol in different groups.

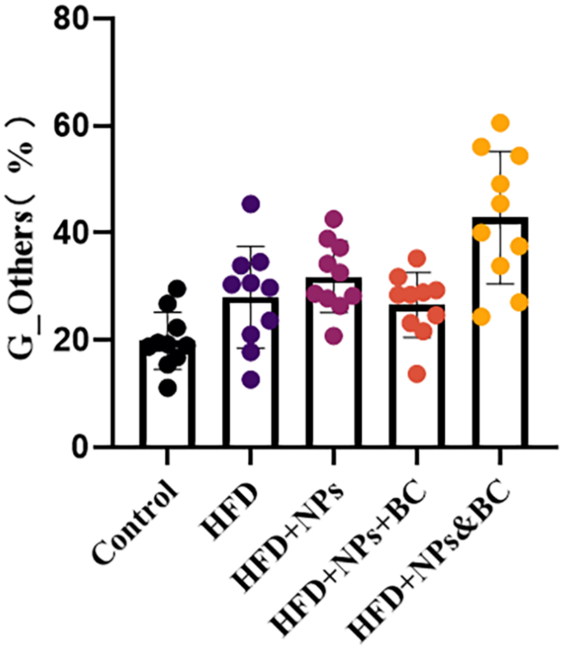

Figure S2 The sum of the other microorganisms at the genus level.
